# ASF (a Compound of Traditional Chinese Medicine) in the treatment of patients with alcohol dependence: Study protocol of a randomized, double-blinded, placebo-controlled clinical trial

**DOI:** 10.1097/MD.0000000000023899

**Published:** 2020-12-24

**Authors:** Xianting Liang, Xiaoyu Hu, Xia Zhang, Hongfang Fu

**Affiliations:** a Hospital of Chengdu University of Traditional Chinese Medicine; b Chengdu University of Traditional Chinese Medicine, Chengdu, China.

**Keywords:** abstinence, addiction withdrawal treatment, alcohol dependence, ASF, randomized controlled trial, relapse, traditional Chinese medicine

## Abstract

**Background:**

Alcohol dependence is one of the biggest problems facing public health worldwide. Currently, it is an under-diagnosed and under-treated disease. Even when given treatments for addiction withdrawal, over 2/3 of patients who have undergone abstinence-oriented treatment will relapse in the first year. Therefore, it is necessary to find an efficacious way to prevent and treat alcohol dependence. ASF (a Compound of Traditional Chinese Medicine) has proven to inhibit the formation and expression of ethanol-induced behavioral sensitization and the development of conditioned place preference in mice. As an empirical prescription for abstinence from alcohol, ASF has long been used in clinical patients. However, the effect of ASF in humans has not yet been investigated. The purpose of this study is to evaluate the efficacy of ASF for patients with alcohol dependence.

**Methods:**

The effect of ASF will be studied in a randomized, double-blinded, placebo-controlled clinical trial. 82 outpatients and inpatients will be recruited and randomly assigned to treatment with either ASF or placebo for 6 weeks as a complement to cognitive behavioural therapy. The primary endpoints are the changes in the average daily alcohol consumption of the 2 groups before and after treatment and comparison of the scores of the psychological craving self-rating scale during the courses of treatment of 2 groups. The secondary endpoints include abstinence rates of the 2 groups during the follow-up period, days without consumption, and changes of Short Form Health Survey (SF-36) scores in 2 groups before and after therapy.

**Discussion:**

This study is the first randomized controlled trial to investigate ASF in the treatment of alcohol dependence. ASF is likely to be a new and effective drug for the treatment of alcohol dependence developed from natural products with a low incidence of side effects or toxicity.

**Trial Registration:**

Registry number: ChiCTR2000039397.

## 1 Introduction

Harmful drinking has been listed by the World Health Organization as the fifth leading risk factor for premature death and disability worldwide, and it is also the main cause of death and disability in developing countries.^[1]^ Alcohol dependence is one of the biggest problems facing public health worldwide. Now, it is an under-diagnosed and under-treated disease, over 2/3 of patients who have undergone abstinence-oriented treatment will relapse in the first year.^[2]^ The pathogenesis of alcohol dependence is closely related to the dysfunction of the reward pathway in the brain. The reward effect of alcohol is mainly transmitted by the “reward pathway” composed of midbrain limbic dopamine neurons and inhibitory gamma aminobutyric acid (GABA) nerve fibers. Normally, a variety of neurotransmitters, including glutamate, GABA, serotonin, glycine, and hypothalamic neurohormones, can regulate inhibitory GABA nerve fibers to maintain the excitability of the midbrain limbic dopamine system.^[3]^ When drinking, alcohol first acts on specific targets, including N-methyl-d-aspartate (NMDA), GABA, glycine, opioid, serotonin, and nicotinic acetylcholine receptors (nAChRs). Then, through the corresponding neurotransmitter or neuropeptide system, alcohol weakens the inhibitory level of dopaminergic pathway in the midbrain margin and increases the level of dopamine release in nucleus accumbens (NAC). Due to alcohol abuse, high-frequency alcohol stimulation can adapt to the structure of reward pathway, leading to dysfunction of the latter and inducing alcohol dependence.^[4]^ How to effectively prevent and treat alcohol dependence caused by excessive drinking has become a studied hot issue in the world. Alcohol dependence is treated with cognitive behavioral therapy (CBT) and pharmacologic treatment.^[5]^ The underlying neuroanatomical underpinnings of alcohol addiction and the therapeutic effect of CBT have not yet been established, although functional MRI research has begun to clarify the neural basis of alcohol dependence. There is a lack of unified and effective drugs for the treatment of alcohol dependence. Three drugs are currently approved by the US Food and drug Administration for the treatment of alcohol dependence, namely, disulfiram, naltrexone, and acamprosate. These drugs can reduce alcohol intake in some patients; however, serious side effects such as related central nervous system symptoms and poor tolerance limit their application range.^[6]^ In view of the low abstinence rate and high recurrence rate of these prescription drugs, there is an urgent need for new and more effective drugs of alcohol dependence. The main driving force for re-drinking after withdrawal treatment is psychological craving. Therefore, reducing the craving for drinking is the key to preventing relapse and treating alcohol dependence. Natural preparations developed from natural products with a low incidence of side effects or toxicity will likely be used to treat alcohol dependence, prevent, and reduce the recurrence. The high-efficiency drugs developed from natural products also show broad market prospects.^[7,8]^

Under the condition of acute short-term drinking, the level of dopamine (DA) in NAC increased rapidly, which is mainly in the positive reinforcing effects of alcohol; while chronic and long-term drinking could cause structural remodeling and down-regulation of reward pathways, mainly showing negative reinforcement of alcohol.^[4]^ Alcohol can inhibit the function of excitatory glutamate receptors and decrease excitatory effect on inhibitory GABA nerve fibers, thereby causing the de-inhibition of dopaminergic neurons in VTA and the increase of DA levels in NAC. That is to say, repeated drinking increases the release of dopamine transmitter in reward pathway, arouses the pleasure of drinkers and produces drinking cravings, which are the positive reinforcement effects of the reward system. Anxiety and other symptoms caused by withdrawal produce craving for drinking, which is in the negative reinforcing effects of reward system, leading to compulsive drinking behaviors.^[9,10]^ Both of them act on the motivation system successively, causing addictive behaviors. ^[11]^ The ideal drugs can reduce alcohol craving by safely and effectively controlling the positive and negative reinforcement effects of alcohol, so as to treat alcohol dependence.

A Compound of Traditional Chinese Medicine (ASF) used in this study comes from the hangover recipe of Professor Xiao-yu Hu. ASF is a compound preparation of traditional Chinese medicine made from Semen Ziziphi Spinosae and Epimedium. Icariin (ICA), the active component of Epimedium, is a flavonoid compound extracted from plants of the genus Epimedium. ICA can regulate the metabolic balance of excitatory amino acid and inhibitory amino acid transmitters by increasing GABA content, decreasing glutamate (Glu) and glutamine content, and reducing Glu/GABA ratio.^[12]^ And total flavone of herba epimediumon (TFE) can increase the release of GABA in the periventricular system of rats and increase the affinity of GABA to the receptor GABAA.^[13]^ Jujuboside A, the active component of Semen Ziziphi Spinosae, increases the expression of GABAA receptors through the blood–brain barrier.^[14]^ The alcohol extract of Semen Ziziphi Spinosae can significantly reduce the expression of N-methyl-d-aspartate receptor 1 and enhance the expression of CABAA receptor 1 in the brain of mice with yin deficiency.^[15]^ The total saponins of Semen Ziziphi Spinosae can reduce the excitatory neurotoxicity in the brain by down-regulating the ratio of Glu and GABA, 2 amino acid neurotransmitters in the old rat model.^[16]^

At present, a variety of drugs for the treatment of alcohol dependence can inhibit the reward effect caused by alcohol and regulate the activity of the reward pathway by acting on GABA receptors at the same time, which has become an important idea of the development of drugs for the treatment of alcohol dependence. Reducing the craving for drinking is the key to preventing relapse and treating alcohol dependence. In view of the fact that both active ingredients can increase the expression of GABAA receptors, whether can we guess Chinese medicine compound ASF by enhancing the inhibition of GABA nerve fibers on the dopamine system in the midbrain limbic region reduce the level of dopamine in NAC caused by alcohol, lower the level of reward effect, and reduce alcohol craving, thereby playing a role in getting rid of alcohol dependence. In China, the application of traditional Chinese medicine has a long history in the prevention and treatment of alcoholism and anti-inebriation.^[17]^ Clinically, ASF has a good therapeutic effect on patients with alcohol addiction. Therefore, in order to verify the efficacy of ASF on patients with alcohol dependence, and to evaluate whether ASF can reduce alcohol craving as its mechanism of action. We have designed a 6-week clinical trial including 82 patients with alcohol dependence. Six weeks are sufficient for the pilot protocol because it is intended to assess the efficacy and tolerability of ASF in the treatment of alcohol-dependent patients.

## 2 Methods/design

### 2.1 Design

**Figure 1:**
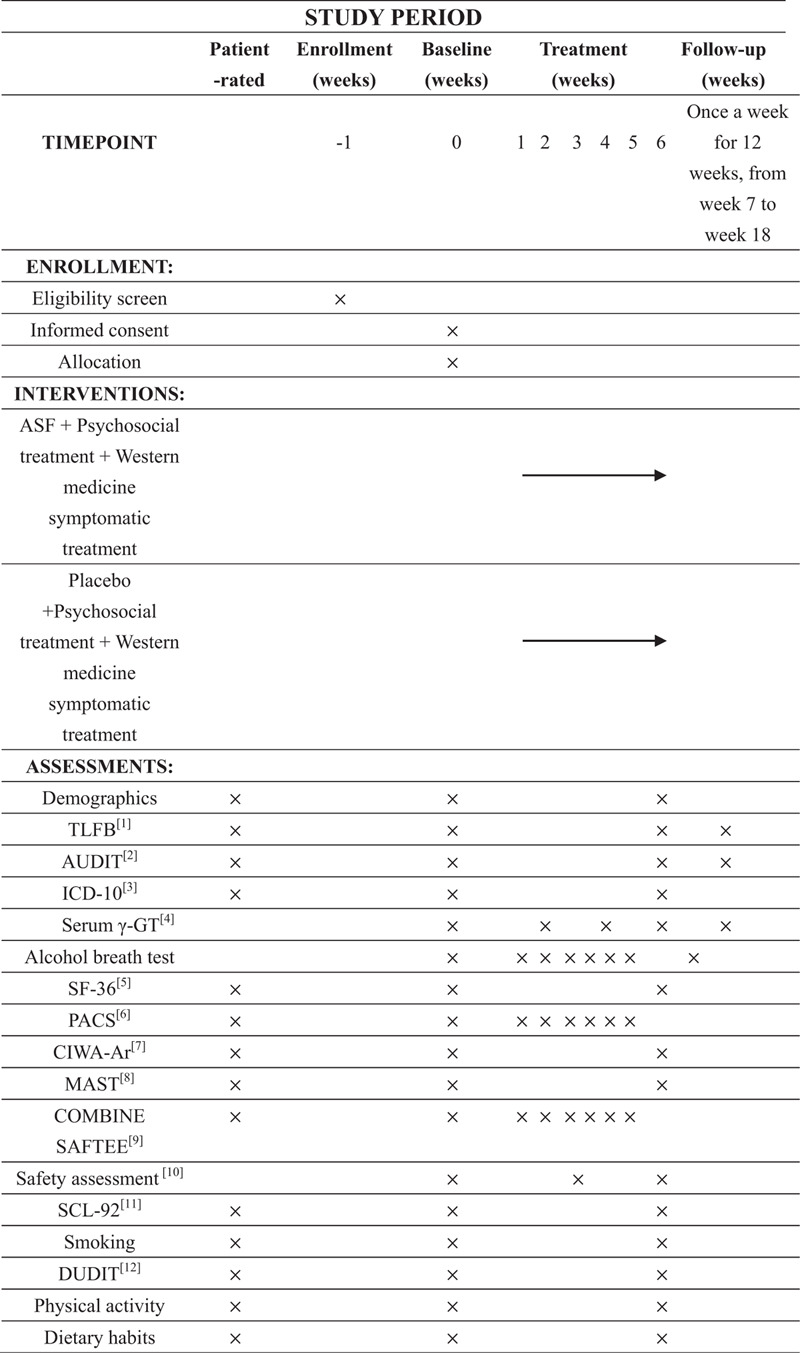
SPIRIT figure: Schedule of enrollments, interventions, and assessments: [1] TLFB = Timeline Follow-back method; [2] AUDIT = Alcohol Use Disorders Identification Test; [3] ICD-10 = International Classification of Diseases, 10th Edition; [4] Serum γ-GT = serum γ-glutamyl transpeptidase; [5] SF-36 = Short Form Health Survey; [6] PACS = Pennsylvania Alcohol Craving Scale; [7] CIWA-Ar = Clinical Institute Withdrawal Assessment of Alcohol Scale, Revised; [8] MAST = Michigan Alcoholism Screening Test; [9] COMBINE SAFTEE = COMBINE Systematic Assessment For Treatment Emergent Events; [10] Safety assessment = blood routine examination, stool routine examination, urine routine examination, kidney function test, liver function test, electrocardiography; [11] SCL-92 = Symptom Checklist; [12] DUDIT = Drug Use Disorders Identification Test.

**Figure 2:**
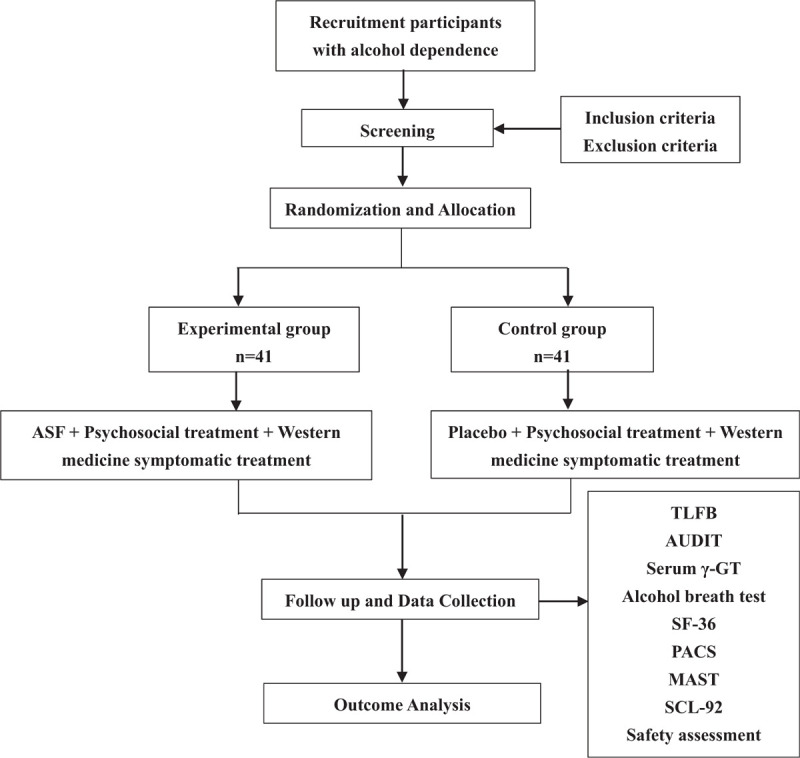
Diagram of the study flow.

The study is a 6-week randomized, controlled, prospective, double-blind clinical trial. After obtaining informed consent, the 82 alcohol-dependent patients recruited will be randomly divided into the ASF group and the placebo group according to 1:1. The study will include a 6-week treatment period and a 12-week follow up time. Both groups of patients will receive psychosocial alcohol therapy and some conventional symptomatic treatments such as liver protection. Besides, for those with severe abstinence symptoms during abstinence, benzodiazepines can be given as appropriate. On this basis, patients in the ASF group will be treated with 1 dose of ASF 3 times a day, while the control group will receive placebo treatment. The purpose is to evaluate the efficacy and tolerability of ASF in treating patients with alcohol dependence and explore the action mechanism of ASF. The protocol is presented in line with the Standard Protocol Items: Recommendations for Interventional Trials 2013 statement (the SPIRIT figure for the schedule of enrollment, interventions, and assessments is presented in Fig. 1; the checklist of SPIRIT is in Supplemental file 1). The diagram of the study flow is shown in Figure 2.

### 2.2 Ethics approval

The study protocol was consistent with the Declaration of Helsinki (Edinburgh 2000) and has been registered in the Chinese Clinical Trial Registry (registration number: ChiCTR2000039397). The protocol was approved by the Medical Ethics Committee of Affiliated Hospital of Chengdu University of Traditional Chinese Medicine (Approval No: 2020SL-011). The design and implementation of the protocol were tracked by the members of the Ethics Committee. All the modifications to the study protocol, CRF, informed consent form, and others will be approved by the Ethics Committee. All the modifications will be updated on the Chinese Clinical Trial Registry.

### 2.3 Recruitment

The patients will be recruited from the outpatient or inpatient department of the Affiliated Hospital of Chengdu University of Traditional Chinese Medicine. The clinicians participating in the withdrawal treatment unit will conduct preliminary screening of patients in the inpatient or outpatient department, and briefly describe the study to the patients who meet the criteria. If the patients are interested in participating, the clinician will alert the research team. A member of the research team will provide them with a comprehensive oral and written description of the research's aims and procedures and the research team members will understand their rights and responsibilities while participating in the study. Screening examinations only be performed after they have agreed to participate and signed the informed consent form. During screening, the patients will undergo related examinations to make sure that all inclusion criteria and none of the exclusion criteria are met.

### 2.4 Sample size

This study adopts a randomized, controlled, double-blind clinical trial design. Based on data from the study by Johnson et al,^[18]^ where withdrawal rate was 54.5% in the treatment group and16.7% in the control group, with an alpha of 5%, and a power of 90%, the estimated sample size for the 2 groups was 68 patients using the PASS 15 software (34 patients in each group). With an estimated dropout rate of 20%, a total number of 82 patients (41 patients in each arm) will be needed.

### 2.5 Randomization and allocation concealment

The statistical professionals will number the random numbers generated by SAS software, and number them according to the serial number. According to this randomization code, the random numbers will be divided into an experimental group and a control group. The randomization results will be included into the opaque envelope. The enrolled patients will open the envelopes according to the entry sequence and be randomly divided into 2 groups with 41 patients in each group using the random numbers generated by SAS software. At the same time, the drug package number is generated by computer according to the random number, and the pharmaceutical Department of Affiliated Hospital of Chengdu University of Traditional Chinese Medicine will be responsible for the preparation and packaging of drugs.

### 2.6 Blinding

In order to ensure the consistency of appearance, shape, smell, and specification, Chinese medicine and placebo will be produced, packaged and labeled by the pharmaceutical Department of Affiliated Hospital of Chengdu University of Traditional Chinese Medicine. The statistical professionals unrelated to this study will work out the procedures for designing, distributing, and breaking randomized code lists. The initial parameters about randomized code lists will be sealed in envelopes as confidential data. The emergency letter will be set up in each drug number, including the appropriate drug group. In case of clinical emergency, it can be opened to break randomized code lists. When randomization is broken, researchers, project leaders, and clinical inspectors will be required to participate in the process and record it in detail. Emergency letters will be uniformly recalled after the end of the test. Patients, investigators, and persons performing data analysis will remain blinded from the time of randomization to time of database unlock.

### 2.7 Diagnostic criteria

These patients were diagnosed with alcohol dependence according to the criteria of International Classification of Diseases, 10th Edition (ICD-10) and as alcohol use disorders according to the criteria of Diagnostic and Statistical Manual of Mental Disorders, Fifth Edition (DSM-5).

### 2.8 Eligibility criteria

Inclusion criteria:

1. Age range 18 to 65 years (both included).
2. Diagnosed with alcohol dependence on the basis of the criteria of ICD-10 and DSM-5.
3. No obvious withdrawal symptoms and Clinical Institute Withdrawal Assessment for Alcohol (CIWA-Ar) score < 15.
4. Michigan Alcoholism Screening Test (MAST) score > 7.
5. Informed written consent.

Exclusion criteria:

1. Neuronal degeneration with irreversible process or severe psychiatric disease, for example, a diagnosis of paranoid psychosis, schizophrenia, mental retardation, or bipolar disorder.
2. Concomitant drugs therapy against alcohol dependence, such as naltrexone, disulfiram, nalmefene and acamprosate, or other treatment with any of the drugs within 1 month prior to inclusion.
3. Concomitant pharmacotherapy with psychotropic medications such as antipsychotics, antiepileptics, mood stabilizers, carbonic anhydrase inhibitors, opioid analgesics, or systemic steroids before enrollment (drug elution periods are allowed, depending on the pharmacokinetic characteristics of the drug).
4. Drug Use Disorders Identification Test (DUDIT) score >6 (male), >2 (female), and meeting the substance dependence standards of ICD-10 (except nicotine and caffeine).
5. Psychological treatment for mental disorder within the preceding 3 months.
6. Having more than 4 unsuccessful hospitalizations for alcohol withdrawal in the past.
7. Patients who met the diagnostic criteria of alcohol dependence and had been completely abstinent for more than 2 months.
8. Patients who are allergic to ASF.
9. Pregnant women, those who plan to become pregnant within 3 months and lactating women.
10. Severe physical diseases, including uncontrolled hypertension, cerebrovascular diseases, pulmonary diseases, thyroid diseases, etc (uncontrolled hypertension (systolic blood pressure >180 mm Hg, diastolic blood pressure >110 mm Hg)).
11. Those who are preparing for surgery or organ transplantation recently.
12. Those who cannot conduct the test as required.
13. Severe heart, hepatic and renal dysfunction, or other severely abnormal laboratory tests (cardiac problems defined as decompensated heart failure (New York Heart Association functional class III or IV)), myocardium infarction within the past 12 months and/or unstable angina pectoris; impaired hepatic function (liver transaminases >3 the upper limit of normal); impaired renal function (estimated glomerular filtration rate (eGFR) <60 mL/min); kidney stones; pancreatitis, etc).
14. Participants in other drug trials within the last 4 weeks.

### 2.9 Termination and withdrawal criteria

Participants will be informed of their right to withdraw at any time. If they withdraw, no further training or questionnaire surveys will be conducted. Before the patients agreed to participate, they are told that if they withdraw, the data collected prior to their withdrawal treatment will be retained and used for analysis, unless they explicitly revoke permission to retain the data. And the reason for withdrawing from the study will be recorded in the case report form. The criteria for termination and withdrawal are as follows:

1. Withdrawing informed consent.
2. Treatment failure.
3. Adverse events.
4. Withdrawing from the study in view of the researcher's consideration of the patient's benefit.
5. Unblinding.
6. Being likely to commit suicide or violence.
7. The combination drugs that have a great impact on the clinical trial, affecting the safety and effectiveness of the therapeutic drugs.
8. Being pregnant during treatment.
9. Failure to comply with clinical trial medication, that is, the total amount of medication taken by the patient is less than 75% of the planned treatment amount.

## 3 Interventions

### 3.1 Treatment plan

All patients will receive psychosocial alcohol treatment based on psychological education elements, motivational interviews, and cognitive behavioral therapy, including informing patients about the causes and consequences of alcohol dependence, the necessity of treatment, and how to conduct the treatment of alcohol abstinence. At the same time, family members should be educated about compliance with the treatment plans. Regular psychosocial alcohol treatment was given to abstainers once a week for 6 weeks. According to the abnormal blood chemical features of the patients, the patients will be selectively given corresponding treatment measures such as liver and kidney preservation, correction of electrolyte and acid–base disturbance, effective fluid infusion, vitamin supplementation, etc. The patients with severe withdrawal symptoms during abstinence could be treated with benzodiazepines.

Patients will be randomly assigned to receive ASF or placebo in a ratio of 1:1.

Experimental group (ASF group): On the basis of basic treatment, the patients will take 10 g ASF 3 times daily, dissolved in 150 mL of warm boiling water, for 6 weeks.

ASF used in this test is provided by the pharmaceutical Department of Affiliated Hospital of Chengdu University of Traditional Chinese Medicine (Sichuan, China).

The whole ingredients of ASF are Epimedium 15 g, Semen Ziziphi Spinosae 15 g. One dose every day, 10 g each time, 3 times a day. The herbs in ASF are mixed, processed, filtered, and spray-dried by pressure to form granules, and packaged into single-dose pouches, each weighing 10 g.

Control group: In addition to the basic treatment, the participants will take placebo granules of 10 g each time, 3 times a day. The placebo granules were dissolved in 150 mL of warm boiling water, for 6 weeks.

The placebos in the study are composed of starch and have no active ingredients.

By adding various food pigments, the appearance and taste of the placebo are as close to the real granules as possible.

The drug distribution of the study will be performed by a pharmacist who is not involved in clinical management to preserve the double-blind design. Each time the patient takes the medication, the research nurse will supervise the process and count the number of medication bags taken and not taken.

### 3.2 Outcome measures

Primary outcomes: The primary outcomes are the changes in the average daily alcohol consumption of the 2 groups before and after treatment and comparison of the scores of the psychological craving self-rating scale during the courses of treatment of 2 groups.

Secondary outcomes: The secondary outcomes include abstinence rates of the 2 groups during the follow-up period, days without consumption, and changes of Short Form Health Survey (SF-36) scores in 2 groups before and after therapy.

The researchers will use weekly self-reports during the follow-up period, clinicians’ judgments, and biochemical examinations to determine whether the patient is abstinent. If the patient has drunk no more than 1 cup since the last visit, the GGT level is normal, or has not increased since the last examination, and the respiratory alcohol concentration is less than 0.10 g/L, the patient is judged to abstain from alcohol.

From the first week to the sixth week, vital signs will be monitored daily. The physical examination will be carried out weekly. The respiratory alcohol concentration will be measured once a week, and drinking craving and adverse events will be evaluated weekly at the time of enrollment and at weeks 2, 4, 6, and 7, serum γ-glutamyl transpeptidase (γ-GT) and blood alcohol concentration will be detected. At weeks 0, 3, and 6, the doctors will give the patients electrocardiograms, and urine pregnancy, blood routine and blood biochemical levels will be detected. During the follow-up period, serum γ-GT and respiratory alcohol concentration will be measured once a week.

### 3.3 Questionnaires

At week 0 and week 6, as well as during the follow-up period, the examiner will work closely with the patient to fill in this Timeline Follow Back (TLFB) schedule for the past month, based on the alcohol diary collected weekly. The TLFB has been widely evaluated and tested,^[19]^ and in previous studies,^[20]^ it has been proved to have high retest reliability. The data collected by TLFB will be applied to evaluate the effects on the primary outcomes and secondary outcomes.

Baseline data for the Clinical trial, the Alcohol Use Disorders Identification Test (AUDIT) questionnaire, the TLFB interview, and the diagnostic instruments or questionnaires for diagnosis of alcohol addiction will be collected routinely for all patients. At week 0 and week 6, as well as during the follow-up period, the questionnaires will be conducted including: demographics (age, gender, marital status, occupation), alcohol consumption (TLFB), withdrawal symptoms (CIWA-Ar), alcohol addiction (MAST), craving (Pennsylvania Alcohol Craving Scale (PACS)), drinking behaviors (AUDIT), health status (SF-36), psychopathology (Symptom Checklist (SCL-92)),drug use (DUDIT), and adverse events (COMBINE Systematic Assessment For Treatment Emergent Events (COMBINE SAFTEE)).

### 3.4 Safety assessment

ASF has a long history in Chinese traditional medicine and is widely used in hospitals. The experimental dose is within the recommended range, which is based on the People's Republic of China Pharmacopeia (2015 edition). The vital signs (blood pressure, pulse, body temperature, respiration) of patients will be measured regularly. Physical examinations will be carried out, and blood biochemistry will be monitored regularly. Adverse reactions of the 2 groups will be monitored by the modified COMBINE SAFTEE, a structured instrument for collecting adverse events adapted for clinical studies in the alcoholism field.

### 3.5 Compliance

During the treatment phase, the researchers will record the type, frequency, and dose of any drug received by the patient during the hospitalization, monitor the patient's medication compliance ((drug bags distributed-returned bags)/total number of prescription bags) and other medications taken during the treatment period, and copy any relevant clinical records made at the time of admission. Another researcher who does not participate in the training management and is blinded to the treatment allocation of the patients will contact follow-up interviews including abstinence and TLFB, during the follow-up period after treatment. According to the intention-to-treat principles, patients who withdraw from treatment will still be contacted by researchers for follow-up before completing the 6 weeks of treatment, unless they withdraw from the study.

### 3.6 Adverse events

All the adverse events that occur during the trial will be examined and documented in this study. The adverse events, which refer to any unintended and undesirable signs (such as abnormal laboratory test results), diseases or symptoms that occur after treatment in the process of clinical trial, do not necessarily have causal relationships with this treatment. The adverse events will be recorded through observation or patient complaints. The incidence of adverse events between the 2 groups will be examined according to occurrence frequency. Once any serious adverse event occurs, the intervention should be stopped immediately. According to China Food and Drug Administration (CFDA) Standard Operating Procedures (SOPs), the intervention time, relationship with the drug, severity, and actions taken will be recorded in detail. In addition, serious adverse events should be reported to the Ethics committee within 24 hours.

### 3.7 Data management and quality control

All the records in the CRFs are filled out and collected by trained and qualified researchers. After the CRF is completed, the original data recorded won’t be changed, even if any changes are made. The clinical examiner will review all the completed CRFs. A medical statistician will guide data entry and management. In order to ensure the accuracy of the data, 2 data managers will input and proofread the data independently. Once the established database is reviewed, it will be locked by key researchers and statistical analysts. The locked files or data will not be changed and will be submitted to the statistics team for statistics and analysis. The scientific research department of the Affiliated Hospital of Chengdu University of traditional Chinese medicine will review the data in the middle of the study.

### 3.8 Statistical analysis

Data analysis will be performed using the SPSS 18.0 statistical software package. Categorical variables will be compared by use of chi-square analysis. Continuous variables between groups will be analyzed by independent sample *T* test. The repeated measure of analysis of variance (ANOVA) will be used to analyze the differences in the group. For all reported values will be expressed as mean ± SEM. The log-rank test will be applied to compare the cumulative probability functions. The Cox proportional hazards model will be applied to assess the hazard ratio of achieving 12 weeks of abstinence and the Kaplan–Meier method will be used to calculate the cumulative probability function of reaching 12 weeks of abstinence. The changes of desire measured by PACS will be compared at baseline and at week 6 through the *T* test. The changes of desire and resistance during the study will be analyzed by using mixed linear regression analysis. All statistical tests will be two-sided tests, and *P* < .05 indicates that the difference is statistically significant.

## 4 Discussion

At present, the important brain regions for studying alcohol dependence include ventral tegmental area (VTA)-NAC-prefrontal cortex (PFC). These brain regions are also the last pathways of reward effect caused by addictive substances.^[3]^ The drugs that are more effective in treating alcohol dependence are likely to come from natural products.^[7,8]^ Relevant animal experiments showed that the Chinese traditional medicine compound ASF can inhibit expression and development of behavioral sensitization induced by ethanol and the formation of conditioned place preference in mice. The inhibitory effect of ASF on behavioral sensitization is partly due to the effect of ASF on the midbrain limbic neurotransmitter system, including the decrease of DA and Glu levels and the increase of GABA content.^[21]^ Therefore, it can be inferred that ASF can prevent compulsive drug behaviors, drug seeking behaviors, and relapse behaviors of alcohol-dependent patients after withdrawal, and have an intervention effect on alcohol dependence. This study provides a certain experimental basis for the intervention mechanism of ASF on alcohol addiction.

However, other mechanisms of ASF against alcohol addiction need to be further studied. Negative emotions such as anxiety, irritability, and physical discomfort may occur in alcohol-dependent patients during abstinence, and the negative emotions can induce craving pathways. Psychological craving is an important factor that causes the relapse of alcohol dependence after abstinence. Negative emotions such as anxiety mean early recurrence and increase the risk of recurrence.^[22–25]^ At present, one of the main goals in the treatment of alcohol dependence is to minimize the recurrence rate. The noradrenergic system in the brain is closely related to anxiety. Under normal circumstances, the noradrenergic system can make people keep a certain level of vigilance. When it is stimulated by alcohol, caffeine and other substances, it will cause people to become excessive excitement, and then appear obvious symptoms of fear and anxiety.^[26]^ Semen Ziziphi Spinosae can treat the anxiety-like behaviors of rodents during alcohol withdrawal by normalizing the noradrenergic nervous system in the hippocampus, thus reducing relapse.^[27]^ On the other hand, long-term and excessive alcohol intake can activate the hypothalamic–pituitary–adrenal (HPA) axis, promotes the hypothalamus to secrete corticotropin releasing factor (CRF), and release adrenocorticotropic hormone (ACTH), which leads to the increase of ACTH and cortisol (CORT) levels in peripheral blood. It has been found that CORT can enhance the activity of dopamine neurons in the midbrain limbic system and increase the release of dopamine neurotransmitters from axon terminals.^[28,29]^ CORT can enhance the reward effect by affecting the central dopamine system.^[30]^ In addition, psychological craving induced by alcohol-related factors can lead to increased concentrations of neuroendocrine hormones such as CORT, norepinephrine (NE), ACTH, and adrenaline (AD) in peripheral blood.^[31]^ It was found that the HPA axis of stress system is related to psychological craving.^[32]^ Therefore, for the treatment of alcohol dependence, timely adjustment of the function of HPA axis system in patients with alcohol dependence can reduce the individual's psychological craving for alcohol and prevent relapse. It is found that icariin significantly reduced the levels of serum CRF and cortisol in CMS rats, and icariin can reduce the high reactivity of CRF system in HPA axis stress loop.^[33]^ Therefore, we speculate that Epimedium can affect the HPA axis system by reducing the high responsiveness of the CRF system, thus reducing psychological craving and ultimately reducing the relapse after alcohol withdrawal.

In summary, whether the traditional Chinese medicine compound ASF made of Epimedium and Semen Ziziphi Spinosae can reduce relapse by reducing alcohol craving. This study is the first randomized controlled trial to study the effect of ASF on alcohol dependence. The traditional Chinese medicine compound ASF is likely to be a new and effective drug for the treatment of alcohol dependence developed from natural products with a low incidence of side effects or toxicity.

## Acknowledgments

The authors are grateful to Dr Xuke Han for her assistance and valuable advice.

## Author contributions

**Conceptualization:** Xiaoyu Hu, Xia Zhang, Hongfang Fu.

**Investigation:** Xia Zhang, Hongfang Fu.

**Resources:** Hongfang Fu.

**Supervision:** Xiaoyu Hu, Xia Zhang, Hongfang Fu.

**Writing – original draft:** Xianting Liang.

**Writing – review & editing:** Xianting Liang, Xiaoyu Hu.
